# Ecological interactions are a primary driver of population dynamics in wine yeast microbiota during fermentation

**DOI:** 10.1038/s41598-020-61690-z

**Published:** 2020-03-18

**Authors:** Bahareh Bagheri, Florian Franz Bauer, Gianluigi Cardinali, Mathabatha Evodia Setati

**Affiliations:** 1 South African Grape and Wine Research Institute, Department of Viticulture and Oenology, Stellenbosch University, Stellenbosch, ZA-7600 South Africa; 2 Section of Applied Microbiology - Department of Plant Biology and Agri-Environmental Biotechnology - University of Perugia Borgo, Perugia, Italy

**Keywords:** Applied microbiology, Microbial ecology

## Abstract

Spontaneous wine fermentation is characterized by yeast population evolution, modulated by complex physical and metabolic interactions amongst various species. The contribution of any given species to the final wine character and aroma will depend on its numerical persistence during the fermentation process. Studies have primarily evaluated the effect of physical and chemical factors such as osmotic pressure, pH, temperature and nutrient availability on mono- or mixed-cultures comprising 2–3 species, but information about how interspecies ecological interactions in the wine fermentation ecosystem contribute to population dynamics remains scant. Therefore, in the current study, the effect of temperature and sulphur dioxide (SO_2_) on the dynamics of a multi-species yeast consortium was evaluated in three different matrices including synthetic grape juice, Chenin blanc and Grechetto bianco. The population dynamics were affected by temperature and SO_2_, reflecting differences in stress resistance and habitat preferences of the different species and influencing the production of most volatile aroma compounds. Evidently at 15 °C and in the absence of SO_2_ non-*Saccharomyces* species were dominant, whereas at 25 °C and when 30 mg/L SO_2_ was added *S. cerevisiae* dominated. Population growth followed similar patterns in the three matrices independently of the conditions. The data show that fermentation stresses lead to an individual response of each species, but that this response is strongly influenced by the interactions between species within the ecosystem. Thus, our data suggest that ecological interactions, and not only physico-chemical conditions, are a dominant factor in determining the contribution of individual species to the outcome of the fermentation.

## Introduction

Alcoholic fermentation of grape juice or “grape must” is commonly initiated by complex microbial consortia composed of oxidative, weakly fermentative and strongly fermentative species^1–4^. During the fermentation process, and dependent on the specific microbiota composition of each must, weakly fermentative yeast species such as *Pichia terricola*, *Metschnikowia pulcherrima* and *Hanseniaspora uvarum* are usually replaced by more strongly fermentative species such as *Lachancea thermotolerans* and *Torulaspora delbrueckii*, while *Saccharomyces cerevisiae* will generally complete the process when alcohol levels are high and oxygen is depleted^5–10^. Many of these weakly and more strongly fermentative species, however, can contribute significantly to the final composition of wine, and impact the sensory perception of the product^11–13^. The contribution of each individual species will depend on its numerical presence and length of persistence throughout alcoholic fermentation. These two parameters, i.e absolute numerical presence, and persistence over time, are affected by parameters such as temperature, sulphur dioxide (SO_2_), pH, osmotic pressure, oxygen, and nutrient availability, and the response of individual species to these parameters has in many cases been investigated, either in single species systems or in co-cultivation with *S. cerevisiae*^11,14–20^. The results indicate that abiotic parameters such as oxygen, temperature and SO_2_ addition strongly affect the fermentation kinetics and yeast population dynamics of the wine fermentation ecosystem^16,21–28^. For instance, low temperatures (10–15 °C) have been shown to enhance the growth of some non*-Saccharomyces* species such as *Starmerella bacillaris*, formerly *Candida stellata*^29^, *L. thermotolerans*^12^ and *Hanseniaspora uvarum*^30,31^ whereas, fermentation at 25–30 °C has been shown to favour the growth of *S. cerevisiae*^28,32^. Addition of 30–50 mg/L SO_2_ as an antiseptic agent to must is a common practice in the wine industry that aims to suppress the growth of undesired yeast and bacterial species such as *Brettanomyces bruxellensis, Lactobacillus* and *Pediococcus* species^19,24,25,32,33^. Previous studies suggest that SO_2_ addition may positively or negatively affect growth of several yeast species during wine fermentation. For instance, SO_2_ addition of between 40–80 mg/L has been shown to negatively affect growth of *H. uvarum*, *L. thermotolerans* and *T. delbrueckii* while supporting growth of *S. cerevisiae*^25,34–36^.

To this end, few studies have attempted to elucidate the impact of ecological interactions between species and of the broader wine ecosystem on the survival and persistence of individual species and their response to temperature and SO_2_. Recent data suggest that such biotic impacts may be significant and that their role in defining population dynamics and fermentation outcomes may have been underestimated. A number of recent articles, for instance, suggest that physical contact between species has significant impact on the performance of these species^37,38^. The data by Rossouw *et al*.^38^ suggest that modifying the level of physical contact will lead to complex changes in population dynamics, either favouring or inhibiting certain species. Other ecological interactions have been reported^12,20,28^. For instance, Alonso del Real *et al*.^28^ showed that cryotolerant *Saccharomyces* yeasts, such as *Saccharomyces uvarum*, compromise the relative fitness of *S. cerevisiae* at lower temperatures. Bagheri *et al*.^8,13^ reported that the presence of certain species can be detrimental to, or may improve, the survival and numerical persistence of other yeast species within the wine ecosystem.

Taken together, the data suggest that biotic selection pressure exerted by other species is a strong driver of population dynamics that has been mostly ignored in literature thus far. The wine ecosystem constitutes likely a good model system to evaluate such impacts since a relatively limited number of yeast species tends to dominate the system, and interactions between these species have occurred within an evolutionarily significant framework. It is therefore highly likely that ecological interspecies interactions within this system would have shaped the genetic makeup and phenotypes of many of these species. Thus, it is essential to ascertain to what degree the specific species composition of any given wine yeast ecosystem will modulate the growth and survival of individual species. The current study aims to evaluate the response of a constructed multispecies wine yeast ecosystem to temperature and SO_2_ addition during alcoholic fermentation of three different grape matrices.

## Materials and Methods

### Yeast consortium and culture conditions

The yeast consortium comprising seven yeast strains obtained from the culture collection of the Institute for Wine Biotechnology (IWBT) and a commercial yeast *S. cerevisiae* Lalvin EC1118 (Lallemand, Canada) was prepared (Table 1) as described by Bagheri *et al*.^8^. The yeast stock cultures were maintained in 20% (v/v) glycerol at −80 °C and were streaked out on Wallerstein Laboratory Nutrient agar (WLN) (Sigma–Aldrich, Spain) when required. The plates were incubated at 30 °C for 3–5 days.

**Table 1:** Strains used in the multi-species yeast consortium.

Species	Strains code	Strains number
*Hanseniaspora vineae*	Hv	Y980
*Pichia terricola*	Pt	Y974
*Starmerella bacillaris*	Sb	Y975
*Candida parapsilosis*	Cp	Y842
*Lachancea thermotolerans*	Lt	Y973
*Saccharomyces cerevisiae*	Sc	EC1118
*Wickerhamomyces anomalus*	Wa	Y934
*Metschnikowia pulcherrima*	Mp	Y981

The strain codes are the abbreviation of the name of each strain.

### Micro-fermentations

Fermentations were performed in synthetic grape juice (SGJ) medium, Chenin blanc (CHN) and Grechetto bianco (GRC) grape juice. The SGJ medium adapted from Henschke and Jiranek^39^, and Bely *et al*.^40^, contained 200 g/L sugars (100 g/L glucose and 100 g/L fructose) and 300 mg/L assimilable nitrogen (460 mg/L NH_4_Cl and 180 mg/L amino acids), and was adjusted to pH 3.5.

**Figure 1:**
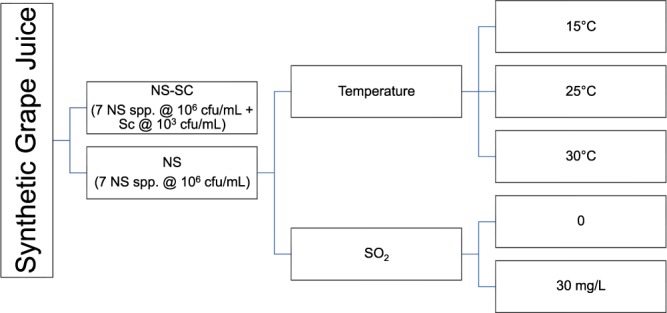
Schematic representation of fermentations conducted in the synthetic juice. Non-*Saccharomyces* species (NS) were either inoculated alone at 10^6^ cfu/mL (NS) or together with *S. cerevisiae* added at 10^3^ cfu/mL (NS-SC).

The effect of temperature (15, 25 and 30 °C) and SO_2_ (30 mg/L) on the dynamics of the yeast consortium (Table 1) was evaluated in the presence (NS-SC) and absence of *S. cerevisiae* (NS), as illustrated in Fig. 1. Non-*Saccharomyces* yeast species were inoculated at 10^6^ cfu/mL while *S. cerevisiae* was inoculated at 10^3^ cfu/mL in 500 mL of juice in NS-SC fermentations whereas the six Non-*Saccharomyces* species were each inoculated at 10^6^ cfu/mL in NS fermentation. Static fermentations were conducted in Erlenmeyer flasks fitted with fermentation locks. The samples were withdrawn at 2-day intervals to monitor the fermentation kinetics. Glucose and fructose were measured, using enzymatic kits, Enzytec™Fluid D-glucose (E5140), fructose (E5120) (Boehringer Mannheim, R-biopharm, Darmstadt, Germany). The fermentations were considered dry when the residual sugar (glucose and fructose) was less than 2 g/L.

For the Chenin blanc and Grechetto bianco fermentations, fifty liters of clarified juice was obtained from a commercial cellar in South Africa and Italy, respectively. Furthermore, viable counts on Wallerstein Nutrient (WLN) agar were performed to enumerate the natural yeast population in each grape juice. Yeast species were isolated and identified by PCR amplification, Restriction fragment length polymorphisms, and sequencing of the ITS-5.8S rRNA gene as described in Bagheri *et al*.^3^. A total of 184 and 212 isolates were obtained from Chenin blanc and Grechetto bianco, respectively.

The Chenin blanc juice was divided into 1.5 L samples and fermentations were conducted in 2 L fermentation bottles in South Africa whereas, in Italy, based on equipment availability, the fermentations were conducted by dividing juice in 500 mL Erlenmeyer flasks. Fermentations were conducted at 15 °C and 25 °C, with 30 mg/L SO_2_ and without SO_2_. Three sets of static fermentations were conducted in triplicate: (i) spontaneous (SP), (ii) inoculated with *S. cerevisiae* EC1118 at 10^3^ cells/mL (EC) and (iii) inoculated with a consortium of 7 non-*Saccharomyces* yeast strains at 10^6^ cells/mL and *S. cerevisiae* EC1118 at 10^3^ cells/mL (NS-SC). Fermentation progress was determined by monitoring CO_2_ release and sugar consumption throughout fermentation. Fermentations were considered complete when residual sugar was less than 2 g/L.

### Analysis of yeast population dynamics

Two mL of synthetic and grape must samples were withdrawn to analyze the population species composition at different stages of the fermentation. Four stages of fermentation including the first day of fermentation (T0) for spontaneous fermentation or the inoculation day (IN) for inoculated fermentations, and the beginning (BF), middle (MF) and end (EF) of fermentations, were selected as sampling points, with BF defined as between 10–20%, MF between 40–60% and EF as more than 95% of sugar consumed, respectively. In case of stuck fermentations, the last sampling point was considered as the end of fermentation. Population dynamics in synthetic must fermentations were monitored using ARISA analysis as previously described in Bagheri *et al*.^8^. We evaluated the efficiency of four different DNA extraction methods as previously described by Siret *et al*.^41^, Garcia-Beneytez *et al*.^42^, Savazzini *et al*.^43^, and Sambrook and Russell^44^. However, low quantity (≤15 ng/uL) and quality (260/280 ratios ≤1) DNA were obtained in all scenarios. An efficient DNA extraction from grape and wine has remained a challenge in grape and wine research; such difficulties have been attributed to the presence of large quantities of polyphenols and polysaccharides^45,46^. In order to eliminate the potential bias introduced by the low quality of DNA, the population dynamics of grape must fermentations were also monitored by direct plating on WL-agar.

### Chemical analysis

Sugar concentrations throughout the fermentation were determined using enzymatic kits, Enzytec^™^Fluid d-glucose (E5140), d-fructose (E5120) (Boehringer Mannheim, R-biopharm, Darmstadt, Germany). The chemical composition of Chenin blanc and Grechetto bianco juice was analyzed by Fourier transform infrared (FT-IR) spectroscopy using the GrapeScan 2000 instrument (Foss Electric, Denmark). Twenty-four hours after the addition of SO_2_ (30 mg/L) in grape juice, the concentration of free and bound SO_2_ was measured, using Ripper method (SM Titrino 702, Metrohm, Switzerland). The yeast species were inoculated 24 hours after SO_2_ addition.

Major volatile compounds were determined using GC-FID as described by Louw *et al*.^47^. Briefly, the extraction was carried out by adding 4-methyl-2-pentanol with a final concentration of 5 mg/L, as the internal standard and 1 mL diethyl ether to each sample. This was followed by sonication for 5 min and centrifugation at 4000 × *g* for 5 min. The ether layer (supernatant) was removed and dried over Na_2_SO_4_. Separation of compounds was done, using a DB-FFAP capillary column (Agilent, Little Falls, Wilmington, USA) with dimensions 60 m length × 0.32 mm i.d. × 0.5 μm film thickness. Furthermore, a Hewlett Packard 6890 Plus GC instrument (Little Falls, USA) equipped with a split/splitless injector and a flame ionization detector (FID) were used for gas chromatography (GC). The gas chromatography was performed under the following conditions: an initial oven temperature of 33 °C for 17 min, followed by an increase in temperature up to 240 °C, for 5 min (12 °C/min). Lastly, three microliters of the diethyl-ether extract were injected at 200 °C in split mode, with a split ratio of 15:1 and the split flow rate of 49.5 mL/min. The column flow rate was 3.3 mL/min, using hydrogen as carrier gas. The detector temperature was 250 °C.

### Statistical analysis

All fermentations and chemical analysis were reported as means ± SD of three repeats. The effect of temperature and SO_2_ on yeast population dynamics and wine aroma was evaluated by conducting an analysis of variance (ANOVA) using the statistical software, Statistica version 13.0 (StatSoft Inc., Tulsa, Oklahoma, USA). The treatments were considered significant should the *p*-values be equal to or less than 0.05. For multivariate data analysis, principal component analysis was performed using the default setting of XLSTAT in Microsoft^®^ Excel (2016).

### Ethical approval

This article does not contain any studies with human participants or animals performed by any of the authors.

## Results

### Grape juice chemical parameters and yeast diversity in grape juice fermentations

Chenin blanc was used for natural grape juice fermentation in South Africa and Grechetto bianco was used in Italy. The Chenin blanc had at a sugar level of 210.7 g/L, pH of 3.37, with a total acidity of 3.23 g/L and 195 mg/L yeast assimilable nitrogen (YAN), while the Grechetto bianco was at a sugar level of 250 g/L, pH 3.17, with a total acidity of 4.41 g/L, and 191 mg/L YAN. The native yeast population in the Chenin blanc juice comprised *S. cerevisiae* (4.85 × 10^3^ cfu/mL), *H. uvarum* (4.20 × 10^3^ cfu/mL), *W. anomalus* (3.34 × 10^3^ cfu/mL), *L. thermotolerans* (2.60 × 10^3^ cfu/mL), and *M. pulcherrima* (2.20 × 10^3^ cfu/mL). In contrast, the Grechetto bianco yeast population consisted of *H. uvarum* (5.20 × 10^4^ cfu/mL), *R. mucilaginosa* (4.8 × 10^4^ cfu/ mL), *P. terricola* (4.30 × 10^4^ cfu/mL)*, M. pulcherrima* (3.70 × 10^4^ cfu/mL) and *S. cerevisiae* (2.10 × 10^3^ cfu/mL). Both grape matrices contained some of the species present in the yeast consortium. However, they were present at levels considerably below the inoculation density of the strains in the yeast consortium.

### Impact of temperature and SO_2_ on fermentation kinetics

The effect of temperature (15, 25 and 30 °C) and SO_2_ (0 and 30 mg/L) on fermentation kinetics was evaluated in the three matrices. The concentration of free SO_2_ after 24 hours of SO_2_ was between 22–32 mg/L in all three matrices.

All fermentations fermented to dryness, with the previously reported exception of the synthetic grape juice inoculated without *S. cerevisiae*^8^. In all cases, the fermentation rate increased proportionally with temperature. In synthetic juice, fermentation at 30 °C (NS-SC-T30-S0) was completed in 20 days, followed by fermentation at 25 °C (NS-SC-T25-S0) and 15 °C (NS-SC-T15-S0) which finished in 22 and 28 days, respectively (Fig. S1A). SO_2_ addition enhanced the fermentation rate in synthetic grape juice, resulting in the NS-SC-T25-S30 completing fermentation within 16 days, compared to the NS-SC-T25-S0 which took 22 days (Fig. S1B). Fermentations performed on Chenin blanc and Grechetto bianco at 25 °C, with 30 mg/L SO_2_ displayed the fastest kinetics whereas the fermentations conducted at 15 °C without SO_2_ showed the slowest kinetics (Fig. S2). Furthermore, fermentations inoculated with EC1118 (EC) were consistently faster than fermentations inoculated with the yeast consortium (NS-SC) and the spontaneous fermentations (SP).

### Impact of temperature and SO_2_ on population dynamics

**Figure 2:**
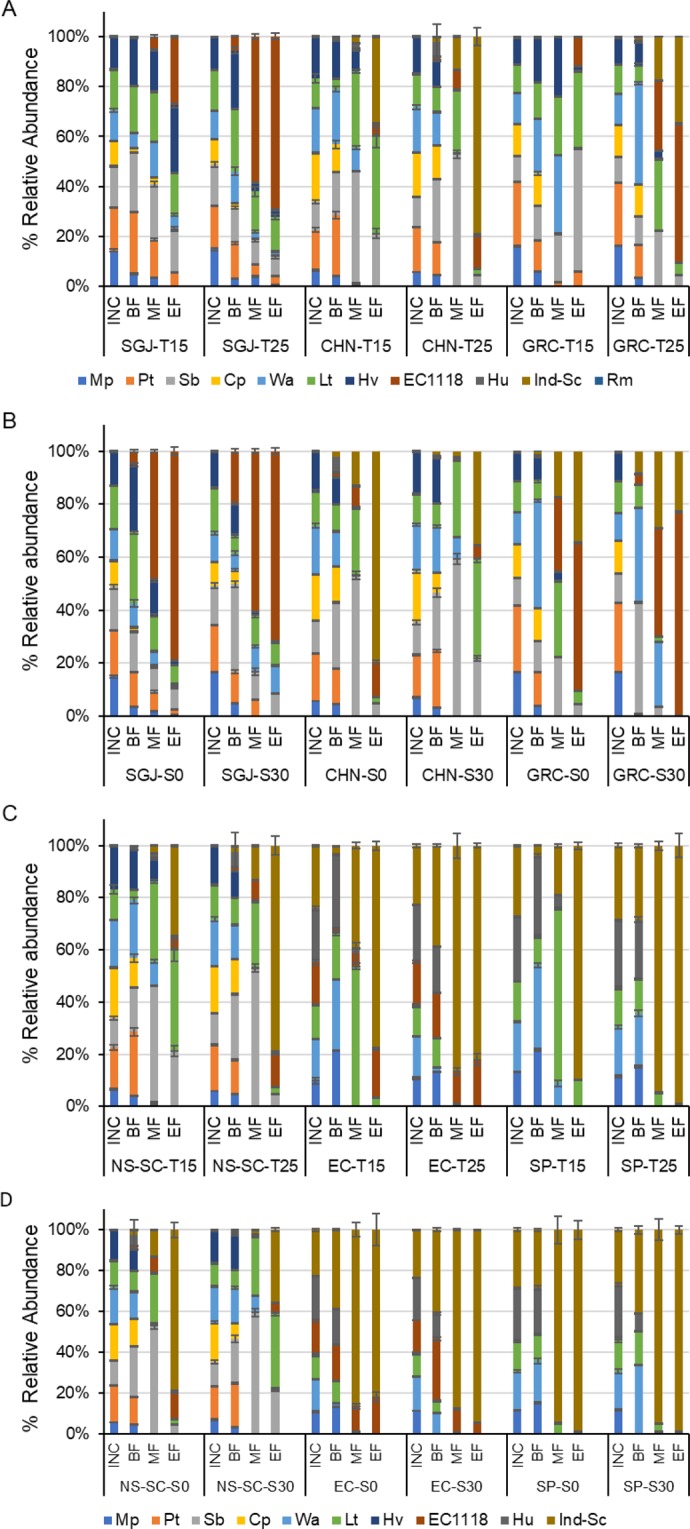
Yeast dynamics in response to temperature (T15 and T25) and SO_2_ (S0 and S30) addition. (**A,B**) show the effect of temperature and SO_2_ on the yeast population dynamics in synthetic grape juice (SGJ), Chenin blanc (CHN) and Grechetto bianco (GRC) at different stages of fermentation. IN shows relative abundance at inoculation, BF, MF and EF is the beginning, middle and end of fermentation with the NS-SC consortium. Fig (**C**) shows the effect of temperature the population dynamics in spontaneous fermentation (SP), the NS-SC consortium and *S. cerevisiae* EC1118 inoculated (EC) Chenin blanc while (**D**) shows the effect of SO_2_. The yeast species monitored were *Metschnikowia pulcherrima* (MP), *Pichia terricola* (Pt), *Starmerella bacillaris* (Sb), *Candida parapsilosis* (Cp), *Wickerhamomyces anomalus* (Wa), *Lachancea thermotolerans* (Lt), *Hanseniaspora vineae* (Hv), commercial *Saccharomyces cerevisiae* (EC1118), indigenous *S. cerevisiae* (Ind-Sc).

Comparison of population dynamics among the three matrices (synthetic grape juice, Chenin blanc and Grechetto bianco) revealed that the population dynamics observed within the NS-SC consortium in Chenin blanc and Grechetto bianco closely resembled the trends observed in the synthetic grape juice fermentations (Fig. 2A,B). For instance, regardless of the grape matrix, non-*Saccharomyces* species including *L. thermotolerans*, *S. bacillaris*, *H. vineae*, *W. anomalus*, and *P. terricola* accounted for 80–90% of the population by the end of all NS-SC-T15 fermentations whereas they made up less than 30% of the population by the end of all NS-SC-T25 fermentations, independently of the presence or absence of SO_2_.

The behaviour of indigenous strains in spontaneous fermentations and fermentations inoculated with EC1118 was similar to that of the same species in NS-SC fermentations. For instance, the behaviour of the indigenous *S. cerevisiae*, *M. pulcherrima*, *L. thermotolerans* and *W. anomalus* in the Chenin blanc SP fermentation was similar to that of the same species in the NS-SC inoculated in synthetic grape juice and in Chenin blanc (Fig. 2C,D). Similarly, the growth of indigenous strains such as *M. pulcherrima* and *P. terricola* in Grechetto bianco fermentations followed similar trends to the inoculated strains in NS-SC fermentations (data not shown). Briefly, *M. pulcherrima* declined rapidly in all fermentations, irrespective of fermentation temperature or SO_2_ addition. However, *M. pulcherrima* strains persisted slightly longer in absence of SO_2_. Results of a two-way ANOVA confirmed that growth of *M. pulcherrima* strains was significantly affected by SO_2_ (Tables S1 and S2). On the other hand, growth of both indigenous and inoculated *W. anomalus* strains was enhanced at 15 °C and in the presence of SO_2_. However, the survival of *W. anomalus* was severely curtailed by high *S. cerevisiae* levels (e.g. EC-T25 and NS-SC-T25). Inoculated and indigenous *L. thermotolerans* strains persisted until the end of fermentation in all fermentation scenarios. However, relative growth of this species was significantly increased at 15 °C in NS-SC and SP treatments (Tables S1 and S2). In contrast, the growth of Ind-*Sc* and EC1118 was enhanced at the higher temperature and in the presence of SO_2_.

The remaining species in the NS-SC consortium *viz*. *S. bacillaris*, *P. terricola*, *C. parapsilosis*, and *H. vineae* maintained similar growth patterns in Chenin blanc and Grechetto fermentations as in synthetic grape juice. Lastly, despite the fact that fermentations were performed under different conditions (T15&T25/S0&S30), the pattern of population dynamics in all NS-SC fermentations remained similar, including the survival of *L. thermotolerans* and *S. bacillaris*, along with *S. cerevisiae*, until middle and end of fermentations and the rapid decline of *M. pulcherrima* and *C. parapsilosis*; *W. anomalus*, *P. terricola*, and *H, vineae* persisted in most cases until beginning and middle of fermentations.

The fermentation conducted in the absence of *S. cerevisiae* (NS) in synthetic grape juice, displayed different patterns of population dynamics, irrespective of fermentation temperature. For instance, *W. anomalus* was the most dominant non-*Saccharomyces* species by middle of NS-T25 and NS-T30, accounting for 65 and 73% of the total population whereas this species accounted for less than 2% of the population by middle of NS-SC-T25 and NS-SC-T30 fermentations. Furthermore, *S. bacillaris* was the most abundant species by the end of NS-T15 whereas *H. vineae* was the most dominant species in NS-SC-T15 (Fig. S3).

### Production of major volatiles

Synthetic grape juice fermentations at 15, 25, and 30 °C produced wines with different aroma profiles. Overall, wine generated from the fermentation at 30 °C (NS-SC-T30) produced the highest level of ethyl esters, mainly due to the high levels of ethyl lactate. However, ethyl caprate and ethyl caprylate were produced at significantly higher concentrations at 15 °C (Table 2). Furthermore, acetate esters were produced at significantly higher levels in the wine generated from the fermentation at 15 °C. In contrast, the fermentation at 25 °C displayed the highest production of higher alcohols (188.9 mg/L), with isoamyl alcohol, 1-propanol, 2-phenyl ethanol, and isobutanol being the main contributors. The production of acetic acid increased proportionally with temperature. However, the NS-SC-T30 fermentation resulted in lower concentrations of total volatile acids, excluding acetic acid. The concentration of some compounds such as ethyl lactate increased proportionally with temperature while others such as 3-ethoxy-1-propanol displayed an inverse relationship (Table 2). Other compounds mainly within the group of volatile acids seemed to not be affected by fermentation temperature

**Table 2:** The concentration of volatile compounds at fermentations conducted at 15 °C (NS-SC-T15), 25 °C (NS-SC-T25) and 30 °C (NS-SC-T30) in the synthetic grape juice.

Compound	NS-SC-T15	NS-SC-T25	NS-SC-T30	p-values	RTS
**Ethyl Esters**					
Ethyl caprylate	0.5 ± 0.07^b^	0.2 ± 0.02^a^	0.2 ± 0.01^a^	0.00	DEC
Ethyl caprate	0.6 ± 0.01^c^	0 ± 0^a^	0.3 ± 0.04^b^	0.00	NP
Ethyl lactate	3.2 ± 0.09^a^	5.6 ± 0.06^ab^	9.5 ± 0.07^b^	0.02	INC
Diethyl succinate	0.1 ± 0.01^a^	0.6 ± 0.13^b^	0^a^	0.00	NP
∑ Esters	4.5 ± 1.98	6.5 ± 0.21	10.1 ± 0.12	0.00	INC
**Acetate esters**					
Ethyl acetate	98.8 ± 2.87^b^	82.7 ± 4.57^a^	84.3 ± 8.83^a^	0.00	NP
Ethyl phenyl acetate	0.3 ± 0.01^a^	1 ± 0.1^b^	0.6 ± 0.03^ab^	0.00	NP
2-Phenylethyl acetate	1 ± 0.1^b^	0.8 ± 0.03^a^	0.7 ± 0.05^a^	0.00	NP
Isoamyl acetate	0.2 ± 0.06^a^	0.6 ± 0.04^b^	0.2 ± 0.05^a^	0.00	NP
∑ Acetates	100.4 ± 3.04	85.2 ± 4.74	86 ± 8.96	0.00	DEC
**Alcohols**					
Isoamyl alcohol	71.9 ± 4.38^a^	100.3 ± 4.81^b^	78.2 ± 5.12^a^	0.01	NP
2-Phenyl ethanol	16.2 ± 2.16^a^	22.5 ± 3.13^b^	18.7 ± 1.24^ab^	0.00	NP
Isobutanol	13.7 ± 6.54^a^	15.9 ± 0.35^a^	20.9 ± 0.8^b^	0.00	INC
Butanol	0.2 ± 0^a^	0.8 ± 0.09^b^	0.7 ± 0.04^b^	0.00	NP
Propanol	20.4 ± 2.89^a^	46.5 ± 6.03^b^	23.3 ± 2.10^a^	0.00	NP
3-ethoxy-1-propanol	3.9 ± 0.32^b^	2.6 ± 0.27^a^	2.3 ± 0.28^a^	0.00	DEC
∑ Higher alcohols (no methanol)	126.6 ± 16.29	188.9 ± 14.68	144.3 ± 9.58	0.03	NP
**Volatile acids**					
Acetic acid	411.5 ± 3.88^a^	614.3 ± 8.95^ab^	693.2 ± 39.65^b^	0.00	INC
Propionic acid	1.7 ± 0.1^b^	0.9 ± 0.04^a^	1.2 ± 0.02^a^	0.01	NP
Isobutyric acid	0.5 ± 0.03^a^	0.8 ± 0.02^b^	0.7 ± 0.04^ab^	0.00	NP
Butyric acid	0.9 ± 0.07^b^	2.2 ± 0.06^c^	0.7 ± 0.01^a^	0.00	NP
Iso-valeric acid	1 ± 0.03^b^	0.8 ± 0.19^a^	1.2 ± 0.01^b^	0.00	NP
Valeric acid	0.4 ± 0.55^a^	1.1 ± 0.03^b^	0.5 ± 0.18^a^	0.00	NP
Hexanoic acid	1.3 ± 0.85^b^	1.2 ± 0.17^b^	0.7 ± 0.2^a^	0.00	NP
Octanoic acid	1.9 ± 1.29^b^	1.7 ± 0.01^b^	1.2 ± 0.3^a^	0.00	NP
Decanoic acid	1.6 ± 0.18^c^	0.9 ± 0.01^a^	1.4 ± 0.02^b^	0.00	NP
∑ Volatile acids without acetic acid	9.7 ± 3.1	9.9 ± 0.53	8 ± 0.77	0.01	NP
**Ketones**					
Acetoin	7.8 ± 0.57^b^	2 ± 1.07^a^	13.8 ± 2.09^c^	0.00	NP

Values are represented in mg/L ± standard deviations. A response pattern of each compound to increasing temperatures (15, 25 and 30 °C) is indicated in a response to temperature (RTS) column. “INC” indicates an increase in concentration of a compound, “DEC” displays a decrease in concentration of a compound in response to increasing temperatures whereas a compound that represent no increasing or decreasing pattern is presented with no pattern (NP). Superscript letters denote statistical differences (p < 0.05). Different letters in the same row indicate significant differences in the compound concentration between the three fermentation temperatures.

**Figure 3:**
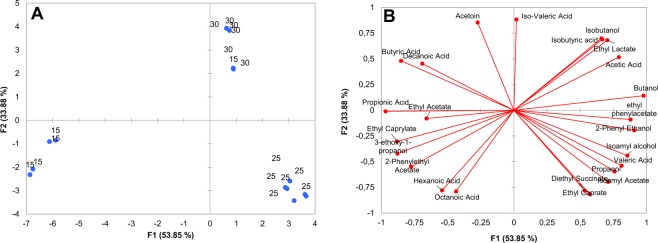
Principal component analysis showing the observations plot (**A**) and loadings plot (**B**) for the major volatile compounds accumulated at the end of fermentation by the NS-SC consortium in synthetic grape juice at 15, 25 and 30 °C.

Principal component analysis was applied to all quantifiable major volatiles to determine the compounds that drive the differences between wines as a function of different temperatures. PC1 and PC2 explained 53.85% and 33.88% of the variance (Fig. 3). The PCA plot indicated that the wine produced at 15 °C (NS-SC-T15) was mainly associated with fatty acids (hexanoic acid and octanoic acid), esters (2-phenylethyl acetate, ethyl acetate, and ethyl caprate and ethyl caprylate) as well as 3-ethoxy-1-propanol. In contrast, wine produced at 30 °C (NS-SC-T30) was characterized by higher alcohols (1-butanol and isobutanol), short chain fatty acids (isobutyric acid, isovaleric acid, and acetic acid), acetoin and ethyl lactate. NS-SC-T25, on the other hand, was mainly associated with esters (ethyl phenylacetate, isoamyl acetate, and diethyl succinate) and alcohols (isoamyl alcohol, 2-phenyl ethanol, and 1-propanol) as well as butyric acid. The results of a one-way analysis of variance (ANOVA), indicated that with the exception of pentanol and ethyl butyrate and, the production of all other major volatiles was significantly affected by temperature.

Chenin blanc grape juice fermented under different temperature and SO_2_ conditions produced different aroma profiles. A two-way ANOVA confirmed that the production of some major volatiles, mainly within the group volatile acids (propionic acid, butyric acid, hexanoic acid, octanoic acid), higher alcohols (iso-butanol, 2-phenyl ethanol, and isoamyl alcohol), few esters (isoamyl acetate and ethyl lactate), and acetoin was significantly affected by temperature. For instance, ethyl lactate was produced at a significantly higher concentration in NS-SC-T25 (25.9 mg/L) compared to NS-SC-T15 (0) whereas isoamyl acetate was significantly produced at a lower concentration in NS-SC-T25 (66.3 mg/L) compared to NS-C-T15 (76.6 mg/L). The production of some compounds such as ethyl lactate, isoamyl alcohol, 2-phenyl ethanol, and acetic acid was significantly affected with both temperature and SO_2_ addition (Table S3). For instance, the highest concentration of acetic acid was produced in EC-T25-S0 (433.26 mg/L), while the EC-T15-S30 represented the lowest content of acetic acid.

Evaluating the production of major volatiles in Chenin blanc wines produced from the NS-SC consortium, revealed that the production of some compounds followed a similar pattern as observed in the NS-SC synthetic wines. For instance, higher concentrations of some compounds such as ethyl lactate, 2-phenyl ethanol, acetic acid, and total ethyl esters were produced at 25 °C fermentations compared to 15 °C in synthetic grape juice and Chenin blanc (Table 3). Inconsistent patterns in the production of other compounds such as ethyl caprate, propionic, octanoic and decanoic acids were observed between the two matrices. Furthermore, production of some major volatile compounds, such as ethyl lactate, isoamyl acetate, 2-phenyl ethanol and isobutanol, followed a same trend in Chenin blanc NS-SC, EC and SP treatments whereas production of other compounds such as ethyl caprylate, isobutyric acid, and iso-valeric acid represented a different pattern in the SP or EC treatments compared to the NS-SC treatments.

**Table 3:** The concentration of major volatile compounds obtained in wines produced from spontaneous fermentation (SP), *S. cerevisiae* EC1118 inoculated fermentation (EC) and the model consortium comprising 7 non-*Saccharomyces* species and *S. cerevisiae* EC1118 (NS-SC) at 15 and 25 °C (T15, T25), without sulphur (S0) and with 30 mg/L SO_2_ (S30).

Compound	Spontaneous fermentation				Inoculated with EC1118				Inoculated with consortium			
	SP-T25- S0	SP- T25-S30	SP- T15-S0	SP-T15-S30	EC-T25-S0	EC-T25- S30	EC-T15-S0	EC-T15- S30	NS-SC-T25-S0	NS-SC- T25-S30	NS-SC-T15-S0	NS-SC- T15- S30
**Ethyl esters**												
Ethyl caprylate	0.9 ± 0.09^ab^	0.8 ± 0.06^a^	1 ± 0.18^abc^	1 ± 0.07^abc^	1.1 ± 0.02^bcd^	0.9 ± 0.04^ab^	1.2 ± 0.15^de^	1.1 ± 0.01^cde^	2 ± 0.03^g^	1 ± 0.01^ef^	1.5 ± 0.04^f^	1.3 ± 0.05^de^
Ethyl caprate	0.6 ± 0.14^ab^	0.6 ± 0.02^a^	0.6 ± 0.06^a^	0.5 ± 0.12^a^	0.9 ± 0.06^cde^	0.5 ± 0.13^a^	0.7 ± 0.25^abc^	0.5 ± 0.07^a^	1.3 ± 0.09^f^	1 ± 0.08^def^	1.1 ± 0.1^ef^	0.8 ± 0.06^bcd^
Ethyl lactate	8.5 ± 0.05^b^	8.5 ± 0.05^b^	0^a^	0^a^	28.1 ± 5.06^c^	8.4 ± 0.09^b^	0^a^	0^a^	25.9 ± 1.42^c^	9.2 ± 0.5^b^	0^a^	0^a^
Diethyl succinate	0.4 ± 0.02^ab^	0.4 ± 0.02^ab^	0.3 ± 0.01^ab^	0.4 ± 0.04^ab^	0.5 ± 0.05^bc^	0.5 ± 0.06^abc^	0.5 ± 0.13^bc^	0.2 ± 0.02^a^	0.4 ± 0.01^ab^	0.4 ± 0.01^ab^	0.7 ± 0.25^c^	0.5 ± 0.13^bc^
Ethyl butyrate	0.2 ± 0.02^a^	0.2 ± 0.02^a^	0.3 ± 0.04^bc^	0.3 ± 0.01^bcd^	0.2 ± 0.01^a^	0.3 ± 0.02^a^	0.3 ± 0.02^cd^	0.4 ± 0.04^fg^	0.3 ± 0.01^bcd^	0.3 ± 0.011^bcd^	0.4 ± 0.03^g^	0.4 ± 0.01^dfg^
Ethyl hexanoate	0.8 ± 0.03^a^	0.7 ± 0.03^a^	0.9 ± 0.17^bc^	1 ± 0.01^bc^	0.8 ± 0.03^ab^	0.9 ± 0.03^ab^	1.1 ± 0.01^cd^	1.3 ± 0.04^d^	1 ± 0^b^	0.9 ± 0.04^ab^	1.2 ± 0.06^d^	1.1 ± 0.09^d^
∑ Esters	11.7 ± 0.33	11.5 ± 0.18	3.3 ± 0.46	3.3 ± 0.25	31.9 ± 5.23	11.7 ± 0.37	4.1 ± 0.56	3.7 ± 0.18	31.1 ± 1.56	13.4 ± 1.44	5.1 ± 0.48	4.2 ± 0.34
**Acetates**												
Ethyl acetate	39.8 ± 1.08^ab^	37.6 ± 1.84^a^	36.3 ± 1.79^a^	36.1 ± 0.78^a^	34.3 ± 3.93^a^	40.3 ± 1.36^ab^	35.5 ± 0.52^a^	38.2 ± 3.11^ab^	66.3 ± 3.75^b^	93 ± 15.40^c^	76.6 ± 6.08^c^	47.1 ± 8.72^ab^
2-Phenylethyl Acetate	0.8 ± 0.04^a^	0.7 ± 0.01^a^	0.6 ± 0.11^a^	0.7 ± 0.01^a^	0.7 ± 0.07^a^	0.9 ± 0.1^ab^	0.7 ± 0.01^a^	0.7 ± 0.04^a^	1 ± 0.06^ab^	1.8 ± 0.86^c^	1.6 ± 0.67^bc^	1.1 ± 0.48^abc^
Isoamyl acetate	2.2 ± 0.17^abc^	2 ± 0.05^a^	2.4 ± 0.54^abcd^	2.7 ± 0.02^bcd^	2.1 ± 0.13^ab^	2.7 ± 0.24^bcd^	2.8 ± 0.12^cd^	3.1 ± 0.32^de^	2 ± 0.06^a^	2.2 ± 0.08^abc^	3.6 ± 0.68^e^	3 ± 0.3^d^
∑ Acetates	43.1 ± 1.36	40.5 ± 1.98	39.8 ± 2.46	39.8 ± 0.85	37.4 ± 4.13	44.3 ± 1.87	39.5 ± 0.68	42.6 ± 3.73	69.5 ± 3.88	97.3 ± 16.42	82.2 ± 7.48	51.6 ± 9.54
**Alcohols**												
Isoamyl alcohol	187.6 ± 4.1^d^	170.9 ± 3^cd^	146.6 ± 4.72^abc^	154.49 ± 3.14^bcd^	161.5 ± 5.5^bcd^	198.6 ± 5^d^	161 ± 2.19^bcd^	181.4 ± 6.35^cd^	113.4 ± 3.70^a^	177.5 ± 13.98^cd^	138.7 ± 4.45^abc^	129.8 ± 4.11^ab^
2-Phenyl ethanol	41.8 ± 2.41^gh^	37.5 ± 0.84^fgh^	23.8 ± 7.71^cd^	26.1 ± 0.67^cd^	35.7 ± 6.23^efgh^	46 ± 8.09^h^	27 ± 2.44^cdef^	31.1 ± 4.37^efd^	16.1 ± 0.54^ab^	33.4 ± 3.08^efg^	14.2 ± 1.30^a^	19.5 ± 5.62^abc^
Isobutanol	29.6 ± 1.56^bd^	25.7 ± 1.08^bcd^	17.9 ± 4.02^a^	17.5 ± 2.50^a^	22.9 ± 2.29^abc^	29 ± 3.44^bd^	18.4 ± 2.18^ac^	19.1 ± 3.47^ac^	28 ± 5.26^bd^	32.9 ± 2.31^d^	23.6 ± 4.86^abcd^	17 ± 2.91^a^
1-Butanol	0.7 ± 0.05^ab^	0.7 ± 0.04^ab^	0.6 ± 0.05^a^	0.6 ± 0.0^a^	0.7 ± 0.0^ab^	0.7 ± 0.07^ab^	0.6 ± 0.0^a^	0.7 ± 0.09^ab^	0.9 ± 0.0^c^	0.9 ± 0.1^c^	0.8 ± 0.04^c^	0.6 ± 0.0^a^
1-Propanol	10.6 ± 0.48^a^	10 ± 0.3^a^	11.3 ± 1.30^ab^	10.5 ± 0.64^a^	9.8 ± 0.33^a^	9.1 ± 0.39^a^	9.8 ± 0.27^a^	9.4 ± 0.37^a^	16 ± 0.91^c^	14.5 ± 1.55^b^	13.5 ± 0.48^b^	10.3 ± 0.54^a^
Hexanol	1.2 ± 0.03^ab^	1.2 ± 0.04^a^	1.2 ± 0.06^ab^	1.2 ± 0.03^ab^	1.2 ± 0^ab^	1.2 ± 0.04^ab^	1.3 ± 0.01^b^	1.3 ± 0.04^b^	1.2 ± 0.02^ab^	1.3 ± 0.03^ab^	1.2 ± 0.04^ab^	1.2 ± 0.05^ab^
3-ethoxy-1-propanol	2.8 ± 0.1^ab^	2.8 ± 0.08^ab^	2.8 ± 0.14^a^	3 ± 0.07^bcd^	2.9 ± 0.06^abcd^	2.9 ± 0.02^abc^	2.9 ± 0.03^abc^	3.1 ± 0.05^de^	3.2 ± 0.06^e^	2.9 ± 0.04^abcd^	3 ± 0.04^bcde^	3 ± 0.02^cde^
∑ Higher alcohols (no methanol)	274.6 ± 8.73	249.1 ± 5.38	204.4 ± 18.00	213.6 ± 17.91	234.9 ± 14.41	287.7 ± 17.05	221.2 ± 7.12	246.4 ± 14.74	179 ± 10.49	263.7 ± 21.09	195.4 ± 11.21	181.7 ± 13.25
**Volatile acids**												
Acetic acid	300.6 ± 4.94^a^	296.9 ± 21.47^a^	285.8 ± 4.55^a^	302.4 ± 16.38^a^	433.4 ± 3.09^b^	261 ± 15.38^a^	265.3 ± 8.73^a^	257.1 ± 10.12^a^	428.8 ± 2.36^b^	272.5 ± 17.11^a^	266.4 ± 9.89^a^	292.0 ± 12.42^a^
Propionic acid	1.2 ± 0.02^bc^	1.1 ± 0.03^abc^	0.6 ± 0.02^a^	1.1 ± 0.03^abc^	1 ± 0.01^abc^	1.1 ± 0.01^abc^	1.1 ± 0.03^abc^	1.1 ± 0.02^abc^	1.1 ± 0.01^abc^	1.5 ± 0.41^c^	1.1 ± 0.04^abc^	0.9 ± 0.02^ab^
Isobutyric acid	2 ± 0.1^b^	1.8 ± 0.04^b^	1.1 ± 0.22^a^	1.1 ± 0.05^a^	1.8 ± 0.28^b^	2.2 ± 0.42^b^	1.2 ± 0.14^a^	1.2 ± 0.16^a^	1 ± 0.02^a^	2.2 ± 0.12^b^	0.8 ± 0.1^a^	1.1 ± 0.12^a^
Butyric acid	1.2 ± 0^ab^	1.1 ± 0.02^a^	1.3 ± 0.14^cde^	1.3 ± 0.06^cde^	1.1 ± 0.03^ab^	1.2 ± 0.05^ab^	1.4 ± 0.04^bcd^	1.4 ± 0.09^ef^	1.3 ± 0.04^bcd^	1.2 ± 0^abc^	1.6 ± 0.05^f^	1.4 ± 0.01^e^
Iso-valeric acid	1.6 ± 0.07^ef^	1.5 ± 0.01^def^	1.2 ± 0.23^bc^	1.2 ± 0.02^bcd^	1.5 ± 0.19^cdef^	1.8 ± 0.27^f^	1.3 ± 0.15^bcde^	1.4 ± 0.16^cde^	0.8 ± 0.01^a^	1.4 ± 0.05^cde^	0.9 ± 0.05^a^	1.1 ± 0.14^ab^
Hexanoic acid	4.1 ± 0.25^a^	4.1 ± 0.21^a^	4.7 ± 0.52^bc^	4.8 ± 0.09^bc^	4.3 ± 0.02^abc^	4.4 ± 0.09^abc^	5.3 ± 0.08^de^	5.4 ± 0.15^de^	4.9 ± 0.14^cd^	4.2 ± 0.14^ab^	5.7 ± 0.06^e^	5.7 ± 0.13^e^
Octanoic acid	6.5 ± 0.31^ab^	6.2 ± 0.28^a^	7.4 ± 0.73^c^	7.7 ± 0.07^c^	7.3 ± 0.15^bc^	7.4 ± 0.22^bc^	8.5 ± 0.18^ef^	8.9 ± 0.49^e^	7 ± 0.18^abc^	6.6 ± 0.34^ab^	8.3 ± 0.17^de^	8.9 ± 0.57^e^
Decanoic acid	2.5 ± 0.09^bcd^	2.4 ± 0.04^ab^	2.5 ± 0.28^bcd^	2.6 ± 0.12^bcd^	2.7 ± 0.06^bcde^	2.9 ± 0.12^de^	2.9 ± 0.14^cde^	3.1 ± 0.05^e^	2.2 ± 0.03^a^	2.4 ± 0.2^ab^	2.5 ± 0.12^bc^	2.8 ± 0.26^cde^
∑ Volatile acids without acetic acid	19.7 ± 0.85	18.9 ± 0.64	19.1 ± 2.19	20.4 ± 0.46	20.4 ± 0.78	21.5 ± 1.2	22.1 ± 0.77	23 ± 1.16	18.6 ± 0.43	20.1 ± 1.27	21.1 ± 0.59	22.3 ± 1.25
**Ketones**												
Acetoin	0^a^	0^a^	0^a^	0^a^	14.6 ± 5.87^ab^	2 ± 0.03^a^	0^a^	0^a^	11.7 ± 1.95^ab^	24.6 ± 8.16^b^	0^a^	0^a^

Values are represented in mg/L ± standard deviations. Superscript letters denote statistical differences (p < 0.05). Different letters in the same row indicate significant differences in compound concentration across the fermentation modalities.

**Figure 4:**
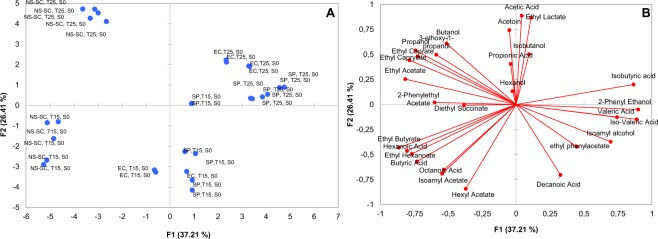
Principal component analysis showing the observations plot (**A**) and loadings plot (**B**) for the major volatile compounds accumulated at the end of fermentation of Chenin blanc. The fermentations were conducted spontaneously (SP), inoculated with EC1118 (EC) or inoculated with the consortium (NS-SC) at 15 °C (T15) and 25°(T25),  mg/L (S30) and without SO_2_ (S0).

Principal component analysis (PCA) was applied on all the major volatiles obtained from the Chenin blanc wines fermented at 25 °C and 15 °C to determine the effect of temperature on the production of major volatiles (Fig. 4). PC1 explained 37.21% of the variation and separated the fermentations which were conducted at 25 °C and 15 °C in the positive and negative dimension of PC, respectively. In contrast, PC2 separated the NS-SC treatments from EC and SP treatments. EC-T25-S0 and SP-T25-S0 made a small cluster and were mainly associated with the short-chain fatty acids (valeric, isovaleric, isobutyric acid and acetic acid), alcohols (isobutanol and 2-phenyl ethanol) and ethyl lactate whereas EC-T15-S0 and SP-T15-S0 formed a group together and were characterized by the high accumulation of acetate esters (hexyl acetate and ethyl phenylacetate), medium chain fatty acids (octanoic acid and decanoic acid) and isoamyl alcohol. On the other hand, NS-SC-T15-S0 was associated with esters including 2-phenyl ethyl acetate, diethyl succinate, ethyl hexanoate, ethyl butyrate, and isoamyl acetate, as well as hexanoic acid. NS-SC-T25-S0 was characterized by high production of ethyl esters (ethyl acetate, ethyl caprate, and ethyl caprylate), alcohols including aliphatic alcohols (butanol and hexanol), propanol and 3-ethoxy-1-propanol as well as propionic acid and acetoin.

## Discussion

The current study evaluated the influence of temperature and SO_2_ on fermentation kinetics and yeast population dynamics in a complex multi-species community. Furthermore, the study aimed to evaluate how changes in population dynamics may affect production of major volatiles in wine. For this purpose, three grape matrices including synthetic juice, Chenin blanc, and Grechetto bianco were selected. Interestingly, some non-*Saccharomyces* species (e.g. *P. terricola* and *M. pulcherrima*) were present in the natural grape juice (indigenous strains) as well as the model consortium (inoculated strains) which allowed us to evaluate whether the effect of temperature and SO_2_ on the growth of yeast species in NS-SC and control fermentations (SP and EC) remains constant.

As for all approaches evaluating impacts and behaviour of multispecies consortia, choices had to be made regarding the specific settings to be evaluated. Consequently, environmental parameters such as pH could not be included although previous studies have shown that pH affects the growth rate, cell biomass and fermentation behaviour of *S. cerevisiae* and non-*Saccharomyces* yeasts in synthetic grape juice^48,49^. Low or high pH can exert chemical stress on yeast cells and depending on the tolerance levels, it would affect yeast population dynamics. Indeed, at 25 °C pH 3.0 and 3.5, a dominance of *S. cerevisiae* with concomitant decline of non-*Saccharomyces* species was reported, suggesting that *S. cerevisiae* is more resilient at these pH levels^50^. Unfortunately, due to logistical constraints we could not investigate this parameter in the current study. Similarly, it would obviously have been useful if additional data on interactions between individual pairings of all of the species, and data on drop-out inoculations (where single species would have been omitted), could have been added. However, this would have represented a very large number of fermentations and would logistically not have been feasible, in particular because we evaluate the impact of the treatments in conditions similar to real industrial wine fermentations.

The data generated with our specific settings nevertheless result in several novel and important insights regarding the behaviour of multispecies yeast ecosystem during fermentation. In particular, they show that, while absolute population numbers of each species are condition dependent, the broad trends in population dynamics are independent of the nature of the specific matrix and of conditions, and are therefore a result of the biotic interactions between the contributing species. The data show that temperature and SO_2_ treatments affected yeast metabolism and growth as reported previously. Fermentations performed at 25 °C and with SO_2_ enhanced the growth of *S. cerevisiae* whereas some non-*Saccharomyces* species persisted longer in the fermentations conducted at 15 °C and without SO_2_. Most of the previous studies have confirmed that non-*Saccharomyces* species can persist longer in the fermentations performed at 10–15 °C^14,28,30,33^. This ability has been linked to modifications in cell wall lipid composition^23,33,51–54^.

Regarding the effect of SO_2_ on population dynamics, it is well documented that antimicrobial activity of SO_2_ is highly pH dependent. At a relatively low pH (<3.5), as was the case in Chenin blanc and Grechetto bianco grape musts, a relatively-higher percentage of molecular SO_2_ is available in grape must^55,56^. Our data confirmed that SO_2_ affects the growth of yeast species in a species- or strain-specific manner. SO_2_ addition displayed inhibitory (*M. pulcherrima*), and supportive (*S. cerevisiae*) effect on the growth of some yeast species while it had a marginal effect on the growth of others (*L. thermotolerans*). Previous studies have reported that SO_2_ affects the growth of organisms in a dose-dependent manner, inhibiting the growth of some species (*P. terricola* and *H. uvarum*) at high concentrations (60 mg/L–100 mg/L) while showing no effect on the growth of other species (*S. bacillaris*) at low concentrations (30–50 mg/L)^24,35,57,58^. Furthermore, the better implantation of *S. cerevisiae* in the yeast consortium in the presence of SO_2_ was in agreement with a previous report by Albertin *et al*.^35^. *S. cerevisiae* has been shown to apply different strategies to adapt to SO_2_^59–61^. However, the supportive effect of SO_2_ at a low dosage (30 mg/L) on the growth of non-*Saccharomyces* species was not reported previously. For instance, *W. anomalus* persisted longer in presence of SO_2_ in all NS-SC and SP fermentations, suggesting that this species is able to detoxify SO_2_ efficiently.

In general, despite the differences among three matrices and the differences in yeast community compositions in NS-SC and the control fermentations (SP and EC), a similar trend of population dynamics was observed in all NS-SC fermentation as well as among NS-SC and control fermentations, even though the strains present in the consortium and grape juice were most probably different. Indigenous non-*Saccharomyces* species Such as *M. pulcherrima, H. uvarum*, and *L. thermotolerans* accounted for a higher proportion of yeast population in SP and EC fermentations compared to the NS-SC fermentation. For instance, indigenous *L. thermotolerans* fermentation, accounted for 53–67% of the total yeast population in the middle of EC-T15 and SP-T15 Chenin blanc fermentations whereas this species accounted for 30% of the population by the middle of NS-SC fermentation. This result can be explained by differences in yeast community composition of SP, EC, and NS-SC which stimulates different yeast-yeast interactions in these communities. Thus, our data underlined the importance of microbial community composition and ecological interaction in wine fermentations.

Another important observation was that despite the differences among the three matrices, synthetic grape juice and the two real grape musts, and the variation in temperature and SO_2_, the survival of *S. bacillaris* and *L. thermotolerans* until middle and end of fermentation was not affected. However, fermentations in absence of *S. cerevisiae* displayed different population dynamics compared to fermentation in presence of *S. cerevisiae*, irrespective of fermentation temperature. The data suggest that the population dynamics in a multi-species consortium is modulated strongly by the response of each species to the presence of other species, rather than by temperature and SO_2_ addition alone. Consequently, the species that evolves better adaptation mechanisms to biotic (competition) stresses present in wine fermentation can outcompete the remaining population and survive longer. Therefore, the dynamics observed in the yeast consortium under different treatments seemed to mainly rely on the ecological interactions, rather than abiotic factors such as temperature and SO_2_ addition.

Regarding the aromatic profiles of wines, our data confirm that the production of some compounds followed a similar trend in all fermentations independently of the microbial consortia, which suggests that the production of these compounds is primary dependent on physical parameters irrespective of the grape matrix. For instance, the production of acetic acid displayed a linear correlation with the fermentation temperature in all of fermentations which is in agreement with a previous report by Torija *et al*.^21^. Since acetic acid is mainly derived from the oxidation of acetaldehyde by aldehyde dehydrogenases (ALDH) as suggested by Remize *et al*.^62^, we can speculate that increase in temperature may have enhanced the activity of ALDH. Secondly, the production of other compounds displayed inconsistent patterns in wines obtained from NS-SC fermentations of synthetic grape juice and Chenin blanc juice, which can be explained by the differences between the two matrices and the differences in the microbial ecosystems. For instance, some yeast-derived volatile compounds such as hexanol and hexyl acetate which require the presence of a grape precursor were only detected in Chenin blanc wine. Lastly, the PC analysis displayed a separation of the fermentations based on temperature (PC1) whereas a further separation was observed between NS-SC fermentations and their respective controls (Fig. 4). This observation suggests that the aromatic profile of wine is mainly affected by the presence of non-*Saccharomyces* species in NS-SC consortium, in spite of the fact that these fermentations were conducted at two different temperatures. Thus, our data show that production of major volatiles is significantly affected by the yeast species composition and the ecological interactions among the species rather than temperature and SO_2._ This result is in agreement with our previous work by Bagheri *et al*.^13^, who confirmed that the aromatic profile of wine is significantly affected by the presence of some non-*Saccharomyces* (e.g. *C. parapsilosis*) in grape juice, irrespective of their rapid declines at an early stage of fermentation.

In conclusion, our data suggest that ecological interactions are a dominant factor in determining the outcome of the fermentation. Furthermore, the data demonstrate that the constructed consortium is a robust model that can be used as a tool to evaluate the contribution of ecological interactions in fermentation dynamics and in the metabolic outcome of the fermentation process.

## Supplementary information

Supplementary information.

## Data Availability

All data generated or analysed during this study are included in this published article and its supplementary information files.
