# Supplementary material for: Ecological interactions are a primary driver of population dynamics in wine yeast microbiota during fermentation
Source: Sci Rep. 2020 Mar 18;10:4911. doi: 10.1038/s41598-020-61690-z (PMC7080794; doi:10.1038/s41598-020-61690-z)
Supplement: Supplementary file 1 — Supplementary information. [file 41598_2020_61690_MOESM1_ESM.docx]

# Supplementary Material Scientific Reports

**Title: Ecological interactions are a primary driver of population dynamics in wine yeast microbiota during fermentation**

**Bahareh Bagheri^1^, Florian Franz Bauer^1^, Gianluigi Cardinali^2^, Mathabatha Evodia Setati^1*^**

^1^Institute for Wine Biotechnology, Department of Viticulture and Oenology, Stellenbosch University, Stellenbosch ZA-7600, South Africa.

^2^Section of Applied Microbiology - Department of Plant Biology and Agri-Environmental Biotechnology - University of Perugia Borgo 20 June, 74. I - 06121 Perugia – Italy.

*Corresponding author information: Dr. Mathabatha Evodia Setati, [setati@sun.ac.za](mailto:setati@sun.ac.za),+27-21-8089203

**Table S1** Results of a two-way ANOVA, indicating the effect of temperature (Temp), sulphur dioxide (SO_2_), and the combined effect of two factors (Tem*SO_2_) on the growth of individual species in fermentations conducted in Chenin blanc. The growth of species with the *p*-values lower than 0.05 was significantly affected by the treatment/combination of treatments. The statistically significant values are represented by red.

| **Species** | **Values** | **NS-SC** | | | **EC1118** | | | **Spontaneous** | | |
| --- | --- | --- | --- | --- | --- | --- | --- | --- | --- | --- |
|  |  | **Temp** | **"SO_2_"** | **Temp**  ***"SO_2_"** | **Temp** | **"SO_2_"** | **Temp**  ***"SO_2_"** | **Temp** | **"SO_2_"** | **Temp**  ***SO_2_"** |
| ***Mp*** | F | 3.35 | 7.00 | 0.44 | 0.00 | 9.82 | 0.00 | 0.00 | 8.86 | 0.09 |
|  | p | 0.07 | 0.04 | 0.50 | 0.92 | 0.00 | 0.97 | 0.95 | 0.00 | 0.75 |
| ***Hu*** | F | 0.10 | 5.18 | 0.06 | 0.19 | 2.76 | 2.46 | 3.68 | 1.55 | 0.66 |
|  | p | 0.74 | 0.02 | 0.79 | 0.65 | 0.10 | 0.12 | 3.68 | 0.54 | 0.66 |
| ***Lt*** | F | 17.74 | 0.00 | 0.20 | 3.08 | 4.60 | 4.10 | 10.55 | 1.45 | 0.38 |
|  | p | 0.00 | 0.93 | 0.65 | 0.08 | 0.03 | 0.04 | 0.00 | 0.23 | 0.53 |
| ***Wa*** | F | 15.36 | 0.00 | 0.03 | 3.66 | 1.63 | 0.64 | 0.34 | 2.72 | 8.16 |
|  | p | 0.00 | 0.93 | 0.86 | 0.06 | 0.20 | 0.42 | 0.55 | 0.10 | 0.00 |
| ***Ind-Sc*** | F | 14.43 | 3.38 | 3.32 | 14.19 | 1.15 | 1.11 | 10.64 | 0.24 | 0.31 |
|  | p | 0.00 | 0.07 | 0.07 | 0.00 | 0.28 | 0.29 | 0.00 | 0.62 | 0.57 |
| ***EC1118*** | F | 20.53 | 2.66 | 2.64 | 15.73 | 0.34 | 0.37 |  |  |  |
|  | p | 0.00 | 0.10 | 0.10 | 0.00 | 0.55 | 0.54 |  |  |  |
| ***Sb*** | F | 10.47 | 0.08 | 1.35 |  |  |  |  |  |  |
|  | p | 0.00 | 0.76 | 0.24 |  |  |  |  |  |  |
| ***Hv*** | F | 8.52 | 1.37 | 0.52 |  |  |  |  |  |  |
|  | p | 0.00 | 0.24 | 0.47 |  |  |  |  |  |  |
| ***Cp*** | F | 1.09 | 2.90 | 1.61 |  |  |  |  |  |  |
|  | p | 0.30 | 0.09 | 0.20 |  |  |  |  |  |  |
| ***Pt*** | F | 5.44 | 0.74 | 0.99 |  |  |  |  |  |  |
|  | p | 0.02 | 0.39 | 0.32 |  |  |  |  |  |  |

**Table S2** The result of two-way ANOVA analysis, indicating the effect of temperature (Temp), sulphur dioxide (SO_2_), and the combined effect of two factors (Tem*SO_2_) on the growth of individual species in the fermentations conducted in Grechetto bianco. The growth of species with the *p*-values lower than 0.05 was significantly affected by the treatment/combination of treatments. The statistically significant values are represented by red.

| **Species** | **Values** | **NS-SC** | | | **EC1118** | | | **Spontaneous** | | |
| --- | --- | --- | --- | --- | --- | --- | --- | --- | --- | --- |
|  |  | **Temp** | **"SO_2_"** | **Temp**  ***"SO_2_"** | **Temp** | **"SO_2_"** | **Temp *"SO_2_"** | **Temp** | **"SO_2_"** | **Temp**  ***"SO_2_"** |
| ***Mp*** | F | 0.07 | 7.10 | 0.04 | 5.71 | 8.20 | 0.00 | 10.14 | 9.76 | 9.77 |
|  | p | 0.78 | 0.04 | 0.83 | 0.02 | 0.03 | 0.98 | 0.00 | 0.00 | 0.00 |
| ***Hu*** | F | 0.33 | 0.31 | 0.00 | 1.03 | 0.97 | 1.30 | 19.42 | 27.05 | 18.43 |
|  | p | 0.56 | 0.57 | 0.98 | 0.31 | 0.32 | 0.25 | 0.00 | 0.00 | 0.00 |
| ***Lt*** | F | 11.74 | 2.16 | 0.53 |  |  |  |  |  |  |
|  | p | 0.00 | 0.14 | 0.46 |  |  |  |  |  |  |
| ***Wa*** | F | 0.09 | 1.71 | 0.01 |  |  |  |  |  |  |
|  | p | 0.76 | 0.19 | 0.89 |  |  |  |  |  |  |
| ***Ind-Sc*** | F | 7.72 | 3.96 | 2.01 | 21.39 | 0.49 | 0.39 | 10.38 | 4.16 | 0.06 |
|  | p | 0.00 | 0.05 | 0.16 | 0.00 | 0.48 | 0.53 | 0.00 | 0.04 | 0.80 |
| ***EC1118*** | F | 5.14 | 3.63 | 2.85 | 11.29 | 0.08 | 1.51 |  |  |  |
|  | p | 0.02 | 0.06 | 0.09 | 0.00 | 0.76 | 0.22 |  |  |  |
| ***Sb*** | F | 0.00 | 15.31 | 0.66 |  |  |  |  |  |  |
|  | p | 0.94 | 0.00 | 0.42 |  |  |  |  |  |  |
| ***Hv*** | F | 6.55 | 7.32 | 0.38 |  |  |  |  |  |  |
|  | p | 0.01 | 0.00 | 0.53 |  |  |  |  |  |  |
| ***Cp*** | F | 0.01 | 2.61 | 0.35 |  |  |  |  |  |  |
|  | p | 0.91 | 0.11 | 0.55 |  |  |  |  |  |  |
| ***Pt*** | F | 0.00 | 1.05 | 0.01 | 0.00 | 4.80 | 0.06 | 8.86 | 16.92 | 8.04 |
|  | p | 0.99 | 0.31 | 0.90 | 0.95 | 0.03 | 0.79 | 0.00 | 0.00 | 0.00 |
| ***Rm*** | F | 0.46 | 1.19 | 0.65 | 0.00 | 0.13 | 0.96 | 2.91 | 5.46 | 2.42 |
|  | p | 0.49 | 0.28 | 0.42 | 0.97 | 0.71 | 0.33 | 0.09 | 0.02 | 0.12 |

**Table S3** The result of a two-way ANOVA analysis, indicating the effect of temperature (Temp), sulphur dioxide (SO_2_), and the combination of treatments on the major volatiles of Chenin blanc wines. The compounds with the p-values lower than 0.05 were significantly affected by the treatment/combination of treatments. The statistically significant values are represented by red.

| **Effect** | **Values** | **"SO_2_"** | **Temp** | **"SO_2_"*Temp** |
| --- | --- | --- | --- | --- |
| **Ethyl acetate** | F | 0.16 | 2.38 | 3.83 |
|  | p | 0.68 | 0.12 | 0.05 |
| **Ethyl butyrate** | F | 0.08 | 69.90 | 0.13 |
|  | p | 0.77 | 0.00 | 0.71 |
| **Ethyl lactate** | F | 19.15 | 130.73 | 19.15 |
|  | p | 0.00 | 0.00 | 0.00 |
| **Ethyl caprate** | F | 5.49 | 3.23 | 0.03 |
|  | p | 0.02 | 0.07 | 0.85 |
| **Ethyl hexanoate** | F | 0.03 | 55.90 | 0.69 |
|  | p | 0.86 | 0.00 | 0.40 |
| **Isoamyl acetate** | F | 0.44 | 46.55 | 1.25 |
|  | p | 0.50 | 0.00 | 0.26 |
| **Diethyl succinate** | F | 7.27 | 0.16 | 2.29 |
|  | p | 0.06 | 0.68 | 0.13 |
| **Ethyl caprylate** | F | 4.63 | 0.11 | 1.11 |
|  | p | 0.03 | 0.73 | 0.29 |
| **Ethyl phenylacetate** | F | 1.70 | 0.03 | 0.24 |
|  | p | 0.19 | 0.84 | 0.62 |
| **1-Butanol** | F | 0.43 | 21.77 | 0.38 |
|  | p | 0.51 | 0.0 | 0.53 |
| **1-Propanol** | F | 6.53 | 1.61 | 0.049 |
|  | p | 0.01 | 0.20 | 0.82 |
| **Isobutanol** | F | 0.00 | 86.47 | 4.65 |
|  | p | 0.97 | 0.00 | 0.03 |
| **2-Phenyl ethanol** | F | 5.16 | 29.20 | 0.60 |
|  | p | 0.02 | 0.00 | 0.44 |
| **Isoamyl alcohol** | F | 4.45 | 7.20 | 2.32 |
|  | p | 0.03 | 0.00 | 0.13 |
| **Hexanol** | F | 0.00 | 0.18 | 2.00 |
|  | p | 0.98 | 0.67 | 0.16 |
| **3-ethoxy-1-propanol** | F | 0.47 | 0.99 | 13.35 |
|  | p | 0.49 | 0.32 | 0.00 |
| **Acetic acid** | F | 16.66 | 21.68 | 29.35 |
|  | p | 0.00 | 0.00 | 0.00 |
| **Propionic acid** | F | 2.25 | 9.75 | 0.02 |
|  | p | 0.13 | 0.00 | 0.86 |
| **Isobutyric acid** | F | 9.18 | 93.82 | 2.61 |
|  | p | 0.00 | 0.00 | 0.11 |
| **Butyric acid** | F | 0.58 | 102.42 | 0.03 |
|  | p | 0.45 | 0.00 | 0.85 |
| **Iso-Valeric acid** | F | 6.15 | 15.45 | 0.41 |
|  | p | 0.01 | 0.00 | 0.52 |
| **Hexanoic acid** | F | 0.21 | 72.21 | 1.27 |
|  | p | 0.64 | 0.00 | 0.26 |
| **Octanoic acid** | F | 0.82 | 86.41 | 4.95 |
|  | p | 0.36 | 0.00 | 0.03 |
| **Decanoic acid** | F | 4.52 | 10.81 | 0.44 |
|  | p | 0.03 | 0.00 | 0.50 |
| **Acetoin** | F | 0.30 | 15.63 | 0.30 |
|  | p | 0.58 | 0.00 | 0.58 |

**Fig S1** Progress curves displaying the kinetics of SGJ fermentations inoculated with the consortium (NS-SC). Figure A represents the fermentation kinetics in the presence of *S. cerevisiae* at 15˚C (NS-SC-T15), 25˚C (NS-SC-T25) and 30˚C (NS-SC-T30) whereas figure B represents the fermentation kinetics in the presence of *S. cerevisiae* with and without sulphur dioxide (NS-SC-T25-S30 and NS-SC-T25-S0).

**Fig S2** Progress curves displaying the kinetics of Chenin blanc fermentations inoculated with the consortium (NS-SC), inoculated with EC1118 (EC) and the spontaneous (SP) fermentation. All fermentations conducted at 15˚C are presented with gray whereas fermentations performed at 25˚C are presented with black. Furthermore, NS-SC, EC and SP fermentations were displayed with circle, square and triangle markers, respectively.

**Fig S3** Dynamics of yeast consortium in the absence and in presence of *S. cerevisiae* at 15˚C, 25˚C, and 30˚C. The figure represents the relative abundance of yeast species throughout NS and NS-SC fermentations (at inoculation time (IN), beginning (BF), middle (MF), end (EF). However, as NS fermentations got stock, the last sampling point was considered as the end point of fermentation.
